# Hypocretin-1 levels in the cerebrospinal fluid of patients with Percheron artery infarction with or without midbrain involvement: A case series

**DOI:** 10.1097/MD.0000000000004281

**Published:** 2016-07-22

**Authors:** Keisuke Suzuki, Tomoyuki Miyamoto, Masayuki Miyamoto, Hiroto Maeda, Kazuya Nokura, Jun Tohyama, Koichi Hirata, Tetsuo Shimizu, Takashi Kanbayashi

**Affiliations:** a Department of Neurology, Dokkyo Medical University, Tochigi, Japan; b Department of Neurology, Dokkyo Medical University Koshigaya Hospital, Saitama, Japan; c Department of Clinical Medicine for Nursing, Dokkyo Medical University School of Nursing, Tochigi, Japan; d Department of Nephrology, Shimonoseki City Hospital, Yamaguchi, Japan; e Department of Neurology, Fujita Health University School of Medicine, Aichi, Japan; f Department of Pediatrics, Epilepsy Center, Nishi-Niigata Chuo National Hospital, Niigata, Japan; g Department of Neuropsychiatry, Akita University School of Medicine, Akita, Japan; h International Institute for Integrative Sleep Medicine (WPI-IIIS), University of Tsukuba, Tsukuba, Japan.

**Keywords:** bilateral paramedian thalamic infarction, hypocretin, Percheron artery

## Abstract

**Background:**

Bilateral paramedian thalamic infarctions (BPTIs) due to artery of Percheron occlusion are known to cause hypersomnia. However, the role of hypocretin-1, a wake-promoting peptide that is located at the lateral hypothalamus, in hypersomnia in these patients remains unclear.

**Methods:**

To clarify the role of hypocretin-1 in hypersomnia in patients with BPTIs, hypocretin-1 levels in the cerebrospinal fluid (CSF) were measured in 6 patients with BPTIs: 2 with rostral midbrain involvement (BPT+RMI) and 4 without midbrain involvement (BPT-MI).

**Results:**

CSF hypocretin-1 levels were decreased in 2 patients with BPT+RMI and were within normal ranges in 4 patients with BPT-MI. Hypersomnia was noted in all the patients. In one BPT+RMI patient, hypersomnia was improved within 2 weeks and decreased CSF hypocretin-1 levels were reversed (acute phase (on day 9), 109.2 pg/mL; chronic phase (at 3 months), 323 pg/mL), whereas another BPT+RMI patient who displayed coma in the acute phase had decreased CSF orexin levels (107 pg/mL) at day 49 and exhibited severe disability.

**Conclusion:**

Hypocretin deficiency was not involved in hypersomnia observed in BPT-MI patients; however, CSF hypocretin-1 levels were reduced in BPT+RMI patients. Reduced CSF hypocretin-1 levels in the chronic phase may possibly predict a poor clinical outcome in patients with Percheron artery infarction.

## 1 Introduction

Hypocretin-1 (orexin-A) is a wake-promoting peptide that is located at the lateral hypothalamus and that plays pivotal roles in maintaining not only wakefulness but also feeding behavior, reward processes, and motivated behaviors.^[1]^ Hypocretin-1 levels in the cerebrospinal fluid (CSF) are consistently low in patients with narcolepsy with cataplexy, which is characterized by excessive daytime sleepiness, sleep paralysis, sleep onset REM period, and hypnagogic hallucination, suggesting that hypocretin deficiency contributes to the development of this sleep disorder.^[2]^ Decreased levels of CSF hypocretin-1 have been described in cases of symptomatic narcolepsy such as hypothalamic lesions due to neuromyelitis optica or multiple sclerosis and tumors.^[3,4]^

The thalamic vascular supply is classically categorized into 4 territories: anterior, paramedian, inferolateral, and posterior territories with variation and overlap. Artery of Percheron (AOP) is an uncommon variant of the paramedian arteries characterized by a single dominant perforating artery supplying the bilateral paramedian thalami.^[5,6]^ Lazzaro et al^[6]^ studied 37 patients with AOP infarction and identified 4 different patterns of AOP infarction: bilateral paramedian thalamic with rostral midbrain (43%), bilateral paramedian thalamic without midbrain (38%), bilateral paramedian and anterior thalamic with midbrain (14%), and bilateral paramedian and anterior thalamic without midbrain (5%). AOP occlusion results in bilateral paramedian thalamic infarction with or without the involvement of the rostral midbrain and/or anterior thalamus likely due to great individual variability in the paramedian arteries. AOP infarction shows a clinical triad including altered mental status, such as coma, drowsiness and hypersomnia; vertical gaze palsy; and memory impairment.^[5–8]^ Sleep/wake disturbances are inevitably observed in patients with AOP infarction from stroke onset or become evident following recovery from coma.^[9]^ The long-term follow-up outcome is generally good, except in cases with midbrain involvement.^[5]^

Narcolepsy–cataplexy associated with decreased CSF hypocretin-1 (167 pg/mL) was reported previously in a 23-year-old man who presented a large hypothalamic stroke following the removal of a craniopharyngioma at the posterior hypothalamus.^[10]^ However, whether hypocretin is involved in hypersomnia in AOP infarction has not been well studied. To clarify the role of hypocretin in hypersomnia in patients with AOP infarction, we assessed CSF hypocretin-1 levels in 6 patients with AOP infarction.

## 2 Patients and methods

Between 2004 and 2015, we identified 6 patients with AOP infarction in whom the CSF hypocretin-1 levels were measured at Akita University School of Medicine.^[11,12]^ The clinical and radiological characteristics of the patients and their relation to the measured CSF hypocretin-1 levels were reviewed retrospectively. The CSF samples were stored at −80°C until analysis. The measurement of hypocretin-1 in the CSF was performed using radioimmunoassay kits (Phoenix Pharmaceuticals, Belmont, CA) as described previously.^[13]^ The intraassay variability was 4.3%, and the detection limit was 40 pg/mL. CSF hypocretin-1 levels of ≤110 pg/mL, 110 to 200 pg/mL, and ≥200 pg/mL were defined as low, intermediate, and normal, respectively.^[13]^ Either the patients or family members provided written informed consent for lumbar puncture and CSF hypocretin-1 measurements. The ethics committee of Akita University School of Medicine permitted our study. We classified the patients with AOP infarction into 2 groups based on the following magnetic resonance imaging (MRI) findings: bilateral paramedian thalamic infarctions (BPTIs) with rostral midbrain involvement (BPT+RMI) and BPTI without midbrain involvement (BPT-MI). Additionally, the involvement of the anterior thalamic nucleus was assessed.^[5]^

## 3 Results

**Figure 1:**
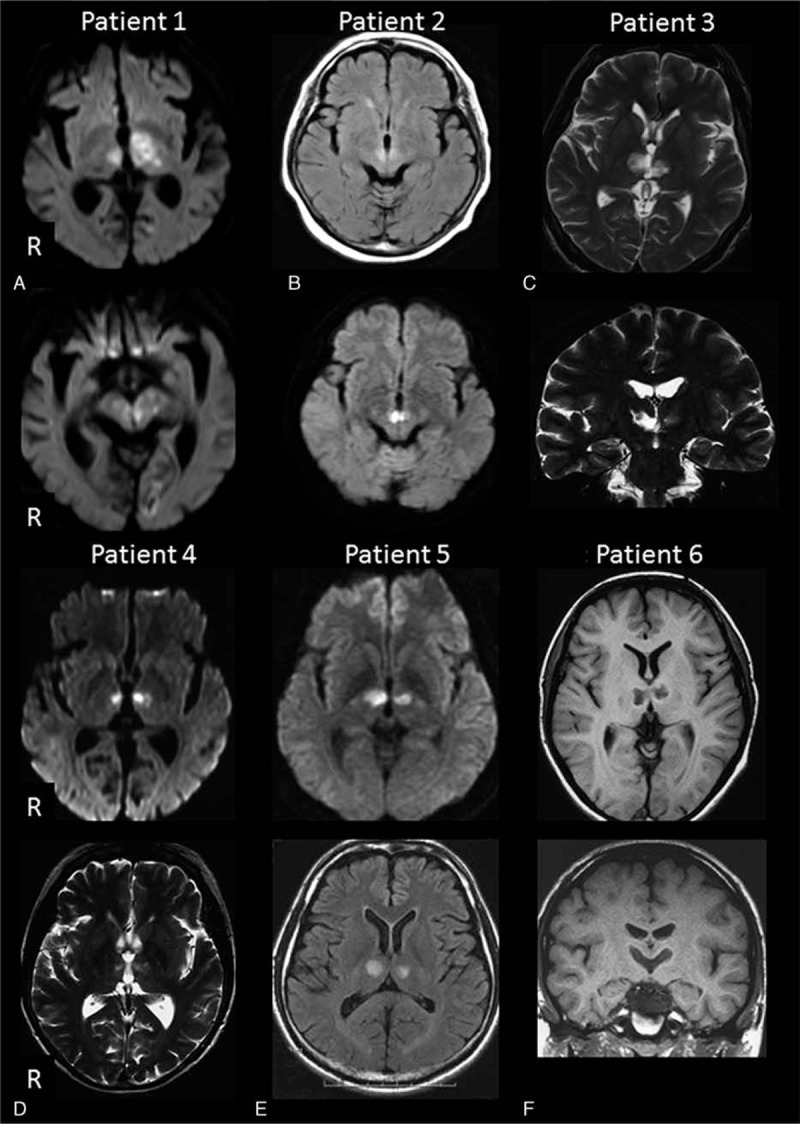
Magnetic resonance images of the patients with artery of Percheron infarctions. A, patient 1; B, patient 2; C, patient 3; D, patient 4; E, patient 5; F, patient 6.

Table 1 shows the clinical characteristics of 6 patients with AOP infarction classified by midbrain involvement. At the initial CSF sampling, all the patients exhibited hypersomnia. Repeated CSF sampling was performed in patient 2 and patient 5 at the chronic stage when hypersomnia symptoms were improved. The MRI findings of each patient are shown in Fig. 1. Two patients with AOP infarctions were classified as BPT+RMI and 4 were classified as BPT-MI. Only one patient with BPT+RMI (patient 1) had anterior thalamus involvement. In patients with BPT-MI, the lesions were restricted to the paramedian thalami.

**Table 1:**
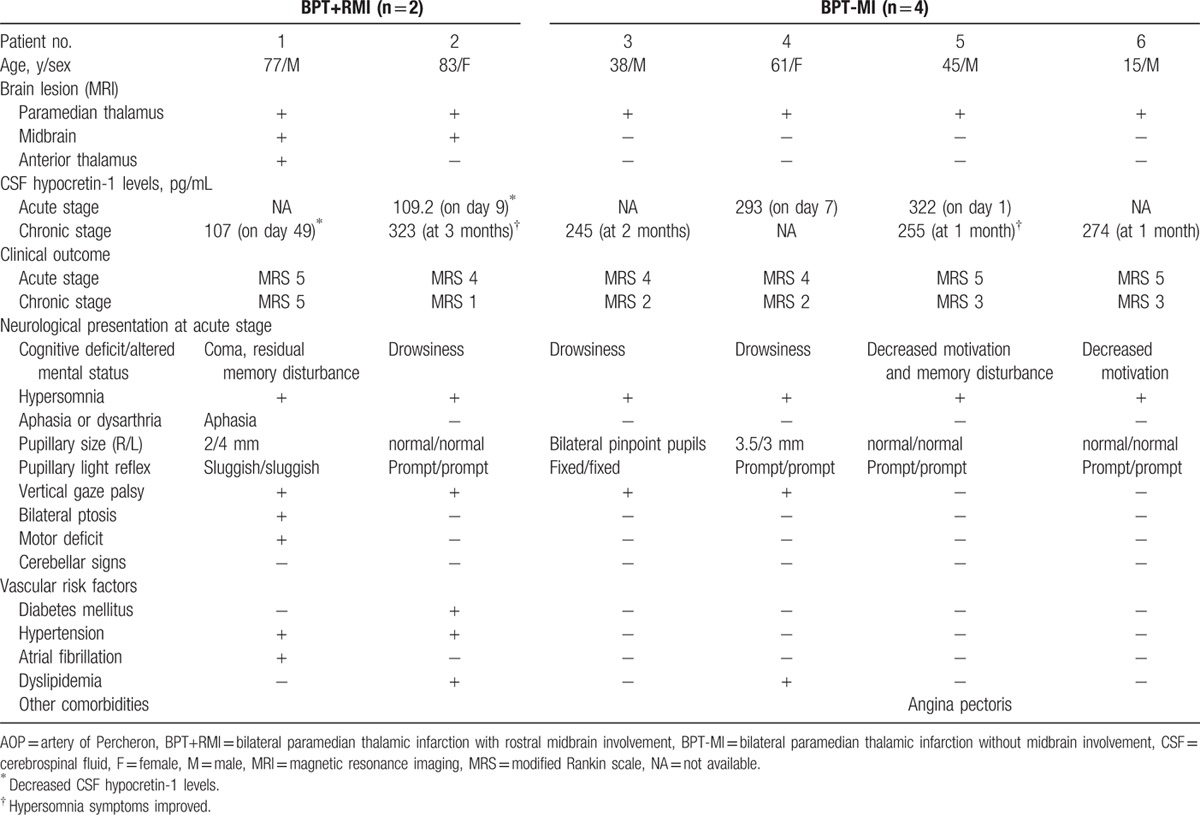
Clinical and radiographic characteristics of the patients with AOP infarction.

Decreased CSF hypocretin-1 levels were observed in 2 of 2 (100%) patients with BPT+RMI, while CSF hypocretin-1 levels were within normal ranges in 4 patients with BPT-MI. All patients with AOP infarction exhibited hypersomnia: patients 2 to 6 showed hypersomnia from stroke onset, whereas patient 1 showed hypersomnia following coma recovery. Vertical gaze palsy was observed in 2 patients (100%) in the BPT+RMI group and in 2 patients (50%) in the BPT-MI group. In 1 BPT+RMI patient (patient 2), hypersomnia was improved within 2 weeks and decreased CSF hypocretin-1 levels were reversed (acute phase, 109.2 pg/mL; chronic phase, 323 pg/mL), whereas another BPT+RMI patient (patient 1) who displayed coma in the acute phase had decreased CSF orexin levels (107 pg/mL) at day 49 and exhibited severe functional disability.

## 4 Discussion

We performed a retrospective analysis of the CSF hypocretin-1 levels in 6 patients with AOP infarctions over a 10-year period. Although likely underestimated, the prevalence of AOP is only in 0.1% to 2% of all strokes and 4% to 18% of thalamic strokes.^[5,6]^ Our primary findings are that the CSF hypocretin-1 levels were not reduced in the patients with BPT-MI but were reduced in the patients with BPT+RMI, who likely had ischemic lesions extending to the lateral hypothalamus. These findings indicate that hypocretin-1 deficiency is not generally involved in increases in sleep needs and hypersomnia observed in patients with AOP infarction when the lesions are restricted to the paramedian thalami. Compared with patients with narcolepsy, who often show undetectable levels of CSF hypocretin-1, decreases in the levels of this neuropeptide were relatively mild in the 2 patients with BPT+RMI.

Hypersomnia in paramedian thalamic stroke is called “pseudo-hypersomnia” and is marked by increased stage N1 and decreased stage N2 and spindles during sleep; however, REM sleep is not affected.^[7,8]^ In the study by Hermann et al,^[8]^ despite improvement in hypersomnia during a follow-up, repeated polysomnography showed no change in the sleep structure except for an increase in the sleep spindle, suggesting that different mechanisms are involved in maintaining wakefulness and sleep structure and that an impaired wakefulness maintenance system is responsible for subjective sleepiness rather than sleep structure changes. In this regard, from our intriguing findings that CSF hypocretin-1 deficiency was not observed in patients with AOP infarction without midbrain involvement, impairment in the wakefulness regulatory systems other than hypocretin-1 could contribute to “hypersomnia” in the acute phase of AOP infarction. Bassetti et al^[14]^ reported normal CSF hypocretin-1 levels (265 pg/mL) in thalamic stroke with subjective sleepiness (Epworth sleepiness scale score, 19/24).

Hypocretin-1-producing neurons are located in the lateral hypothalamus and adjacent perifornical regions, and these neurons heavily project to the rostral brainstem.^[1,15]^ CSF hypocretin-1 levels appear normal in patients with lesions restricted to paramedian thalami due to AOP infarctions because the lesions are not involved in the hypocretin cell bodies. In our study, patient 2 (BPT+RMI) showed reversible subjective sleepiness in association with a reversal of decreased CSF hypocretin-1 levels (acute phase, 109.2 pg/mL; chronic phase, 323 pg/mL), indicating impairment of hypocretin projections, but not of hypocretin cell bodies, during the acute phase. In contrast, patient 1 showed decreased CSF hypocretin-1 levels during the chronic phase (107 pg/mL), displaying residual sleepiness and memory disturbance with a resultant poor functional outcome. Among 12 patients with PTIs (4, bilateral; 4, right; 4, left), clinical outcomes were favorable in the right-sided lesions but were poor in the bilateral and left-sided lesions, which were associated with residual cognitive deficits found in the bilateral and left-sided lesions.^[8]^ Consistent with those study results, patient 1 with anterior thalamus lesions had residual cognitive impairment and poor functional recovery in our study. Additionally, because hypocretin has a central role in maintaining not only the sleep/wake cycle but also reward processes and motivated behaviors,^[1]^ the decreased CSF hypocretin-1 levels in the chronic phase, which resulted from impaired hypocretin cell bodies, could possibly predict a poor clinical outcome in patients with BPT+RMI.

The study limitations include a retrospective setting and a lack of statistical comparison due to the small number of patients examined. We could not correlate the sleep study results with the CSF hypocretin-1 levels in this study. Further prospective studies with larger number of patients will be necessary to investigate the relationship between subjective sleepiness and CSF hypocretin-1 levels, and these should involve sleep studies and hypocretin-1 measurements at multiple points to determine how baseline hypocretin-1 levels predict functional prognosis.

In conclusion, our pilot study showed that hypocretin deficiency was not involved in hypersomnia observed in patients with BPT-MI; however, the CSF hypocretin-1 levels were reduced in patients with BPT+RMI.
